# Are Individual Differences in Reading Speed Related to Extrafoveal Visual Acuity and Crowding?

**DOI:** 10.1371/journal.pone.0121986

**Published:** 2015-03-19

**Authors:** Romy Frömer, Olaf Dimigen, Florian Niefind, Niels Krause, Reinhold Kliegl, Werner Sommer

**Affiliations:** 1 Humboldt-Universität zu Berlin, Berlin, Germany; 2 Universität Potsdam, Potsdam, Germany; University of Copenhagen, DENMARK

## Abstract

Readers differ considerably in their speed of self-paced reading. One factor known to influence fixation durations in reading is the preprocessing of words in parafoveal vision. Here we investigated whether individual differences in reading speed or the amount of information extracted from upcoming words (the preview benefit) can be explained by basic differences in extrafoveal vision—i.e., the ability to recognize peripheral letters with or without the presence of flanking letters. Forty participants were given an adaptive test to determine their eccentricity thresholds for the identification of letters presented either in isolation (extrafoveal acuity) or flanked by other letters (crowded letter recognition). In a separate eye-tracking experiment, the same participants read lists of words from left to right, while the preview of the upcoming words was manipulated with the gaze-contingent moving window technique. Relationships between dependent measures were analyzed on the observational level and with linear mixed models. We obtained highly reliable estimates both for extrafoveal letter identification (acuity and crowding) and measures of reading speed (overall reading speed, size of preview benefit). Reading speed was higher in participants with larger uncrowded windows. However, the strength of this relationship was moderate and it was only observed if other sources of variance in reading speed (e.g., the occurrence of regressive saccades) were eliminated. Moreover, the size of the preview benefit—an important factor in normal reading—was larger in participants with better extrafoveal acuity. Together, these results indicate a significant albeit moderate contribution of extrafoveal vision to individual differences in reading speed.

## Introduction

There are considerable individual differences in reading speed and reading strategies between unimpaired adult readers [1–4]. It is also well-established that reading is facilitated by information in the parafovea about properties of the not-yet-fixated word [5]. If a word can be preprocessed parafoveally during the fixation on the previous word, fixation durations are shorter once the word is foveated (preview benefit). The present study addressed the question whether individual differences in reading speed and preview benefit are related to extrafoveal vision and the crowding effect.

The standard procedure to manipulate preview benefit is the gaze-contingent boundary paradigm [6–8]. In this paradigm, target words are either visible or covered with a mask until the eyes pass an invisible boundary, after which the target word is revealed. In this paradigm, the preview benefit effect is defined as the difference in fixation duration on words that were parafoveally visible versus those that were covered by a mask; it’s size varies between 20 and 50 ms, depending on the type of mask [9]. This effect is a strong contribution to reading speed, considering that fixation durations on single words are merely around 250 ms. Moreover, preview benefit is larger in skilled, as compared to less skilled readers [10, 11]. These findings indicate that extra-foveal information is an important determinant of reading speed.

Vision outside of the fovea (radius of about 2°) [12], that is, extrafoveal vision, has been extensively investigated in vision research [12]. The extrafoveal field can be further subdivided into parafoveal (radius 2 to 5°) and peripheral regions (> 5°). Extrafoveal vision differs from foveal vision among others in terms of reduced acuity and contrast sensitivity [13]. An important phenomenon specific for extrafoveal vision is crowding, the impairment of stimulus identification by flanking stimuli relative to unflanked presentation [14, 15]. As an example, when a letter is presented at an eccentricity of 5° visual angle relative to fixation, it is easily identified, when presented in isolation. The same letter is nearly unidentifiable, when it is flanked by other letters or visual objects. The size of the crowding effect decreases with the distance between target stimulus and flankers, and when the stimulus configuration is closer to the fixation point.

Crowding as a general phenomenon is independent of acuity, contrast etc., as long as these features are above the thresholds for the identification of the visual objects shown in isolation [16]. Moreover, crowding is not only present in letter recognition [17], but also in object [18, 19] and face recognition [20, 21].

Crowding is usually conceived as a problem for stimulus identification at the level of feature integration whereas stimulus detection is largely unaffected [17, 19, 22, 23]. Pelli, Palomares and Majaj [22] argued that in crowding all visual features are extracted but cannot unambiguously be assigned to the target or flanker stimuli. As an alternative to false assignments of features, Greenwood, Bex and Dakin [24] proposed positional averaging as the source of the crowding effect. They suggested that there is positional uncertainty on the location of features. To reduce uncertainty, the position of a stimulus is estimated by averaging across the whole percept (i.e., a triplet of letters). A related approach explains crowding by coarse resolution of attention [25] or unfocused spatial attention [26], also highlighting the role of location.

Independent of the actual underlying mechanism, crowding is determined by the proximity of the flanking stimuli. The impact of spacing on letter and word identification is well established [27]. Therefore crowding is usually manipulated with the critical spacing procedure, which assesses the underlying psychometric function by varying the spacing within letter triplets [22].

Similar procedures as applied to assess crowding are also used to measure the visual span profile [28]. In this paradigm, letter triplets are presented at varying eccentricities across the visual field and must be identified by the participants. The typical visual span profile resulting from this paradigm suggests that letter recognition accuracy seriously drops with eccentricity. This effect can be explained by crowding. Indeed, Pelli, Tillman, Freeman, Su, Berger and Majaj [29] argued that the visual span is effectively the same as the uncrowded window and that the size of this uncrowded window determines reading rate. In particular, if crowding is increased by reducing the spacing between target letters and flanking letters, reading rate drops [27].

According to Pelli’s suggestion the size of a person’s uncrowded window should crucially contribute to his or her individual reading rate and possibly also to the amount of useful information that is obtained parafoveally from not-yet-fixated words (i.e. the preview benefit). However, there are some constraints for such a conclusion. First, experimental manipulations, for example of letter spacing, were usually performed within-participant and correlations across individuals between extrafoveal vision and reading speed have not been reported. The correlations between crowding and reading speed measures reported by Yu, Cheung, Legge and Chung [27], for example, are across condition, but within subjects. Although these correlations were very consistent within each of the five participants, within-subject correlations do not necessarily transfer to correlations across participants. Second, in many of the studies on the relationship between reading speed and crowding measures, the reading situation differed from natural reading in several respects, most importantly by precluding eye movements. Specifically, reading rates were typically determined during word-by-word presentation of isolated words at specified locations within the visual field [28, 30] ([27] for an exception, though without eye-tracking).

The importance of eye movements in reading has long been known [31] and it is widely accepted that reading and the associated eye movement characteristics underlie substantial and consistent individual differences. Findings from passive vision without eye movements therefore do not necessarily generalize to active viewing conditions. Individual differences in eye movements are also found in passive viewing or conditions with little visual stimulation. Notably, they can be separated from individual differences in active exploration, as is the case in natural reading [32, 33]. Hence, as eye movements, such as saccades and regressions, in reading appear to be idiosyncratic and special, individual differences in reading should be assessed in active reading situations with eye movements. The relevance of eye movements for the crowding effect was suggested by Harrison, Mattingley and Remington [34]; these authors reported a reduction in crowding immediately before a saccade and concluded that saccade targets are temporarily released from crowding. This finding was recently replicated for face stimuli [35]. Other studies show that the crowding effect is modulated by shifts of covert spatial attention [36] that accompany eye movements. It is therefore an open question whether the magnitude of a person’s crowding effect—or extrafoveal acuity—predicts an individual’s speed of saccadic reading and the size of the preview benefit.

In order to address this question and to extend previous findings to the individual differences level, the present study assessed the relationship between individual differences in foveal visual acuity and extrafoveal vision (acuity and crowding) and reading time measures, such as reading rate and preview benefit. Accounting for the crucial role of eccentricity and considering, that spacing does not vary in normal reading, we targeted the boundaries of the windows in which isolated and crowded letters can be identified. To that aim, extrafoveal acuity and crowding were measured as individual threshold eccentricities for single and flanked letter identification, using an adaptive procedure. Reading measures were obtained in a separate word list reading experiment with eye-tracking, where in two of three conditions, preview benefit was manipulated with a classic moving-window paradigm [37]. The no-manipulation condition was intended to measure individual differences in overall reading speed under normal conditions.

To assess variability in the speed of self-paced reading, participants were not instructed to read at maximal speed but at their own typical pace. We assumed that standard visual acuity measured at the fovea would not predict individual differences in reading speed, whereas visual acuity and, in particular, crowded letter identification measured in extrafoveal regions of the visual field should do so. Specifically, we expected not only generally faster reading rates, but also larger parafoveal preview benefits for participants with better extrafoveal acuity and a larger uncrowded window.

## Methods

### Participants

Participants were 17 women and 23 men, aged 17 to 44 (*M* = 26.58 years; *SD* = 6.67), who received course credits or money and gave informed written consent. Minors (n = 1, age 17) provided additional consent of their parents. All participants had normal visual acuity (*M* = 1.51, *SD* = 0.37), as measured with the adapative computerized Freiburg Acuity Test (FrACT) [38], which is based on the foveal presentation of Landolt rings in eight different orientations. For 22 of the participants the right eye was dominant, for 16 it was the left eye. For the two remaining participants, ocular dominance could not be unambiguously determined. The study was approved by the institutional ethics committee of the department of psychology of Humboldt University Berlin. This approval includes the testing of minors under extended consent conditions, as conducted. The study was conducted according to the Declaration of Helsinki.

### Overall design and general procedures

The study consisted of two test sessions one week apart. In the first session, a test of uncrowded and crowded extrafoveal vision, the extrafoveal vision assessment (EVA), was administered twice in order to determine the reliability of the test. In addition, visual acuity in the fovea was measured by means of the FrACT. In the second session one week later, the eye-tracking experiment with list reading was conducted. This was followed by a third measurement with the EVA procedure.

### Apparatus

During all experiments, stimuli were presented on a 22 inch CRT monitor (Iiyama Vision Master Pro 510, resolution 1024 x 768 Pixel, refresh rate 160 Hz). Participants were seated at 60 cm distance from the screen, their heads stabilized by the headrest of the eye tracker. During both tests (EVA and word list reading), eye movements were recorded binocularly at a sampling rate of 500 Hz using a table-mounted infrared video-based eye tracker (iView-X Hi-Speed 1250, SensoMotoric Instruments, Teltow, Germany) with an instrument spatial resolution of less than 0.01° and an absolute gaze position accuracy of up to 0.2°. Stimulus presentation and response logging were controlled by *Presentation* Software (Neurobehavioral Systems).

### Extrafoveal Vision Assessment

The EVA is an adaptation of the visual span profile procedure [28]. To measure the span of uncrowded and crowded letter identification in an efficient and reading-relevant manner, we varied horizontal eccentricity of the target letter. As neither of these parameters vary in normal text, letter size was held constant, as well as the target-flanker spacing in the crowded condition. The aim of this procedure was to determine individual differences in the area in which the uptake of extrafoveal information is possible.

#### Stimuli

Letters presented in the EVA were displayed in a monospaced (fixed-width) Courier font a font size of 16 points. At this font size and viewing distance, the center-to-center spacing of adjacent letters is 0.47°, and the pixels of the letter “X” subtend about 0.42° of visual angle horizontally. The target letter on each experimental trial was randomly selected from the 26 capital letters of the alphabet. In the single letter identification task target letters were presented in isolation; in the crowded conditions target letters flanked to the left and right by two additional capital letters that were again randomly and independently selected from the alphabet. In the crowded condition with flanking letters, target-flanker spacing was 0.58° center-to-center, meaning that an additional whitespace of 0.11° was inserted between adjacent letters. During the test, stimuli (single letters or letter triplets) were presentend on the horizontal meridian of the screen. Horizontal eccentricity of the target letters was between 0° (screen center) and ±17.5° to the left or right.

#### Procedure

The EVA realized four test conditions: Single letter identification and flanked letter identification in the left and right visual fields. The four conditions were administered in separate blocks, with the sequence of the four blocks counterbalanced across participants. Before the start of the test, participants were given eight practice trials, two of each condition.

**Fig 1:**
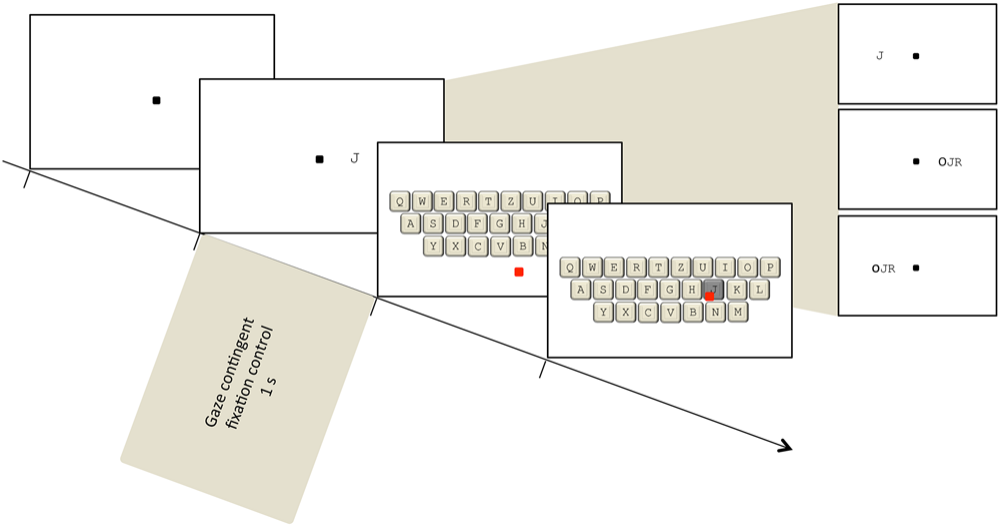
Procedure for the Extrafoveal Vision Assessment (EVA). After a fixation period of 1 s, the target stimulus was presented for 1 s. Horizontal eccentricity of the target stimulus was varied from trial to trial using an adaptive stair-case procedure. The response was given on a digital keyboard using the computer mouse. The right part of the figure depicts the other three experimental conditions: left-hemifield single letter identification, as well as left- and right-hemifield identification of flanked letters.

The trial scheme is illustrated in Fig. 1. Each trial started with the presentation of a central fixation point with a diameter of 0.07°. After a steady fixation interval of 1 s, this was followed by the presentation of the target letter for 1 s on the horizontal meridian of the screen. To control central fixation, binocular gaze position was recorded continuously with the eye tracker. If participants blinked or if the gaze position of either of their eyes deviated clearly from the fixation point (> 1.87° in the horizontal or vertical direction), the trial was automatically aborted with a visual feedback. If participants maintained proper fixation, the presentation of the stimuli was followed by a response screen showing a digital keyboard (see Fig. 1). Participants chose the letter they had identified by selecting the corresponding button on the virtual keyboard, using the computer mouse. Flankers were not enquired. As this test is forced-choice, the procedure only continued once a letter was selected. Participants were instructed to guess in case of a failed identification.

After each response, critical values were computed to determine the horizontal eccentricity of the target stimulus in the subsequent trial. The adaptive staircase procedure PEST [39] was used to converge to the 65% detection threshold for each condition and participant individually. The resulting values will be referred to as threshold eccentricities. The algorithm was set to a starting eccentricity of 300 pixels (10.8°) for the single letter identification and 90 pixel (3.24°) for the flanked letter identification with identical settings for the left and right visual field. The initial step size was set to 10 pixels (0.36°) and the confidence interval for the expected number of hits was set to ±1.5. Additionally, two break-off criteria were applied: If actual step size fell below 2 pixels (0.072°) or if after 21 trials no further change was initiated, the last measured eccentricity was accepted as the threshold value. As the probability of correctly guessing among 26 letters is below 0.04, no correction was applied.

#### Data analysis

For analysis, the measured threshold eccentricities were submitted to a repeated measures Analysis of Variance (ANOVA) with factors extrafoveal vision (single vs. crowded letter), hemifield (left, right), and test repetition (T_1_, T_2_, T_3_). In all ANOVAs, *p*-values are based on Huynh-Feldt adjusted degrees of freedom. Effect sizes are reported as partial eta squared (η_p_^2^). Reliabilities for crowding and single-letter identification are given as Cronbach-α values.

### Reading Task

To measure reading performance, eye movements were recorded in a simplified reading paradigm with lists of unrelated words. One advantage of this paradigm is that it facilitates the creation of stimulus materials for a large number of trials. Furthermore, the paradigm precludes modulating effects of contextual predictability on the preview benefit (because all words are unpredictable) as well as word type and word length effects (all words are content words of medium length). The size of the preview benefit obtained in this paradigm is within the range typically observed in sentence reading studies [40]. Thus, participants read short lists of five German nouns in a self-paced, left-to-right fashion with the task to identify the names of animals contained in some of the lists. To assess individual differences in parafoveal preprocessing, the preview on the upcoming words was systematically manipulated using the moving window paradigm [37].

#### Stimuli

Words presented in the word lists were selected from a pool of 1248 German nouns of lengths between 4–6 letters (*M* = 5.2, *SD* = 0.8). Mean type frequency was 12.3 per million words (*SD* = 38.0), as determined based on the 100-million word DWDS core corpus [41]. In each trial, a list of five words was presented on the horizontal midline of the screen, each word being separated by one empty character space. Across blocks and preview conditions, word frequency was matched. Moreover orthographic similarities between words within the same list were precluded. As required by German orthography, the first letter of each noun was capitalized. In the reading experiment, words were presented in the same monospaced Courier font as in the EVA, but at a slightly smaller font size of 15 points and the default center-to-center spacing, which was 0.43° between adjacent letters. Near the left and right edge of the screen, the word list was flanked by two small black fixation points that were used by the participant to control the reading flow (see below).

#### Procedure

During the experiment, the participant’s task was to read the list of five words and to indicate afterwards whether one of the words had been the name of an animal. This semantic decision was chosen to make sure the participants read all words in the list.

The experiment implemented three preview conditions: 1-word moving window, 2-word moving window, and normal reading. In the normal reading condition, the entire word list was visible throughout the trial without any preview manipulation and masking. In contrast, in the conditions with a 1-word and 2-word window, only the currently fixated word or the currently fixated plus the subsequent word in the list respectively, were visible on any given fixation. In these conditions, the remaining words were covered gaze-contingently with a mask and only uncovered during the incoming saccade. Masks consisted of the letter, x”(in Courier font) with the same length as the corresponding word. This kind of mask provides a stimulus in the parafovea enabling saccade programming, but does not provide orthographic, phonological, lexical or semantic information about the upcoming word. As German nouns always begin with a capital letter, this notation was also applied to the mask, meaning that the word “Frau”, for example, was masked by the string “Xxxx”. The masking and unmasking of words was triggered whenever the participant’s gaze crossed invisible vertical boundaries placed in the center of the empty space between adjacent words. The average latency from the first eye crossing the boundary to the execution of the display change was below 10 ms, meaning that the vast majority of display changes occurred during the saccade (see [40] for details).

To accustom the participants to the reading task, they first read 24 word lists for practice, with 8 lists shown from each of the three preview conditions. The following main experiment consisted of 210 list reading trials, separated into six blocks of 35 trials each. Two blocks of each preview condition (1-word window, 2-word window, and normal reading) were shown to each participant, with the order of blocks counterbalanced across participants. Within each block, the preview condition was held constant. Of the 35 lists in each block, 10 lists (28.6%) contained the name of an animal. Detection of the target item in a given trial changes oculomotor behavior. Specifically, it leads to inflated fixation times on the target word and a tendency to skip the remaining words. In line with our previous experiments with this paradigm, [40] and with previous work using a list-reading task [42] we treated target trials as filler items and excluded them from all analyses. Thus, for each preview condition, eye movement data from 50 lists reading trials entered the analysis.

**Fig 2:**
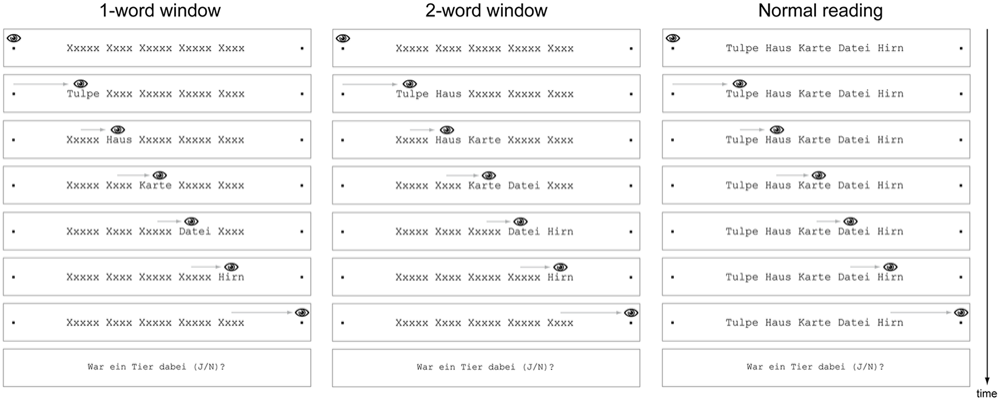
Trial scheme for the list reading task. Participants read short lists of German nouns from left to right with eye movements. Depicted is an example trial in the three preview conditions: 1-word moving window (left), 2-word moving window (middle), and normal reading (right).

Fig. 2 depicts the trial sequence. Each reading trial started with a fixation check on the left fixation point. After a successful fixation, the full list of five words (or placeholder masks) appeared on the horizontal midline of the screen. As the gaze moved across the list, the currently fixated word and—depending on the condition—also the following word was unmasked gaze-contingently. In the normal reading condition, regressive saccades towards earlier words in the list were possible. In contrast, in the moving window conditions, words were remasked and stayed masked once the eyes had left the word in rightward direction for the first time. For this reason, regressive saccades were of no use in these conditions, because they did not reveal the masked word.

To finish reading, participants looked at the right fixation point for 500 ms. This fixation terminated the trial and initiated the presentation of a response screen asking whether or not the name of an animal was contained in the list (“War ein Tier dabei (J/N)?”). The response was given by pressing the left or right mouse button for either yes or no.

Before each block, the eye tracker was calibrated using a standard 9-point grid. Additionally, recalibrations were initiated whenever the automatic fixation check at the beginning of the trial failed, that is, if eye position deviated by more than 0.5° from the left fixation point or if binocular disparity exceeded 0.5°.

#### Data Analysis

After excluding trials with blinks, missing data in the eye-track, or incorrect responses to the animal questions, 95% of the trials (without targets) remained for analysis. In these trials, saccades were detected using the velocity-based algorithm described in Engbert and Mergenthaler [43] (velocity threshold: 5 SD). Small saccades spanning less than one character were considered as part of the fixation. For the assignment of fixation locations, the position of both eyes was averaged. Fixations on inter-word spaces were assigned to the word to the right. Extremely short (< 50 ms, n = 56) or long (>1000 ms, n = 35) fixations were removed.

Across all participants, a total of *n* = 22,290 first-pass reading fixations (excluding fixations following regressive saccades) were detected, corresponding to an average of 185.8 observations per participant and preview condition.

We measured first fixation durations (FFD), gaze durations (GD), and total trial reading durations (TTRD). FFD is the duration of the first fixation on a word, irrespective of whether the word is subsequently refixated. GD is FFD plus the duration of all immediate refixations. In contrast to the fixation-based measures FFD and GD, TTRD was defined as the total time that was spent reading the word list, from the onset of the word list until the participant looked at the fixation point on the right side of the screen. TTRD is therefore a more global measure of reading speed, because it includes the durations of all saccades as well as the durations of those fixations that follow regressive saccades towards previous words in the list (i.e. fixations that occur during re-reading). For analysis, all reading measures were log-transformed to obtain (approximately) Gaussian distributions.

The three eye movement measures were submitted to separate repeated measures ANOVAs on the factor reading condition. The preview benefit was defined as the difference between the 1-word minus the 2-word window conditions, hence a larger preview benefit corresponds to a more positive value. Post-hoc pair-wise comparisons of the different reading conditions were performed by means of *t*-tests, corrected for multiple comparisons according to Bonferroni. To determine the reliability of the reading measures (including the preview benefit), split half reliabilities were computed by separating the reading trials into odd and even.

### Relationships between Extrafoveal Vision and Reading

The relationship between measures of extrafoveal vision and reading behavior was assessed in two ways. In a first set of analyses, we calculated correlations between the eccentricities obtained in the extrafoveal vision test and the reading measures. Because we had directed hypotheses about the relationship between extrafoveal vision and reading behavior, statistical significance of the resulting correlations was assessed with one-tailed tests.

A potential problem with this approach is that several of the measures used in the present study are difference scores that result from the subtraction of two variables. One example is the difference between the identification threshold in the single letter condition and the crowded condition in the EVA. Another important difference score used in the present study is the preview benefit, defined as the difference between the mean fixation duration on parafoveally previewed words and parafoveally masked words. Correlations between such difference measures (and third variables, e.g. reading speed) are usually problematic, because their size is constrained by the reliabilities of the difference measures. Specifically, the reliability of difference scores depends on the internal consistencies of the subtracted measures and on the correlation between the two measures. As the correlation between the two measures increases, the reliability decreases. Hence, reliability of difference scores is usually low (but there are exceptions).

One option to address the problem of a possibly low reliability of within-subject effects (i.e., of differences between experimental conditions) and the low correlation between such within-subject effects is to estimate (co-)variances of these effects as parameters (i.e., variance components and correlation parameters) in a LMM. Essentially, the LMM “corrects” for three possible sources of low reliability of a given subject’s mean (i.e., extreme score, low number of observations, e.g., due to missing values, and large within-subject variance) by shrinking such a subject’s observed mean towards the population estimate [44]. The net effect of these adjustments may be negligible, but can also be quite substantial, for example LMM-based correlation parameters may be of larger magnitude or even of opposite sign than corresponding within-subject correlations [45, 46]. For these models we used the lmer program of the lme4 package [47]. The package is supplied in the R system for statistical computing (version 3.1.1) [48] under the GNU General Public License (Version 3, June 2007). Statistical significance was assessed using (a) likelihood ratio tests and (b) profiling the model parameters. Profiling delivers 95% confidence intervals for model parameters estimating fixed effects, variance components, and correlation parameters [49].

## Results

### Extrafoveal Vision Assessment

#### Experimental Effects

Table 1 displays the mean values and standard deviations of the threshold eccentricities in the single letter and crowding conditions, for each hemifield and test repetition.

**Table 1:** Mean threshold eccentricities (in degrees of visual angle) for the single letter identification and crowding conditions, hemifields, and test repetitions (T_**1**_ to T_**3**_).

Condition	Hemifield	T_1_		T_2_		T_3_		Total	
		*M*	*SD*	*M*	*SD*	*M*	*SD*	*M*	*SD*
Single letter	right	10.79	.80	10.80	.75	10.82	.46	10.80	.56
	left	10.94	.80	11.22	.71	10.84	.66	11.00	.56
Crowding	right	3.26	.46	3.23	.42	3.21	.49	3.23	.40
	left	2.99	.65	2.96	.61	3.02	.53	2.99	.54

ANOVA showed a significant and large main effect of extrafoveal vision, *F*(1, 39) = 9824.21, *p* <. 001, *η*_*p*_^*2*^ = .996, with much smaller threshold eccentricities for crowded as compared to single letter identification. There were no main effects of hemifield and test repetition. However, there was a significant interaction of hemifield and extrafoveal vision, *F*(1, 39) = 28.44, *p* <. 001, *η*_*p*_^*2*^ = .422. Post-hoc analyses within the crowding and single letter conditions revealed significant effects of the factor hemifield with larger threshold eccentricities in the right hemifield for the crowding condition, *F*(1, 39) = 20.24, *p* <. 001, *η*_*p*_^*2*^ = .342, replicating the previously reported left-right- asymmetry of crowding [28, 50]. A reverse effect, larger left than right identification threshold, was found in single letter identification, *F*(1, 39) = 6.58, *p* = .014, *η*_*p*_^*2*^ = .144. In single letter identification, there was also a significant interaction of hemifield and repetition, *F*(1.99, 77.96) = 4.33, *p* = .016, *η*_*p*_^*2*^ = .100. We are not sure why the overall best performance was obtained for detecting single letters in the left hemifield at the second measurement (i.e., 11.22); perhaps there was some condition-specific practice effect. Recall that the second measurement was the second measurement in the first session; the third measurement occurred one week later. It simply may also be a spurious result. Reliability of single letter identification was good, *α* = .81, and reliabitity of crowding was excellent, *α* = .91.

### Reading Task

Table 2 summarizes reading behavior in the three preview conditions. In most of the trials, participants provided a correct answer to the animal question, with error rates of 7.7, 8.4, and 4.6%, for the 1-word window, 2-word window, and normal reading condition, respectively. A repeated measures ANOVA on the error rates revealed a main effect of condition, *F*(2,78) = 17.42, *p* < 0.01, *η*_*p*_^*2*^ = .309. Post-hoc comparisons showed significant differences only between normal reading and the two moving window conditions, *F*(19, 31) ≥ 33.76, *p* <. 001, *η*_*p*_^*2*^ ≥. 325, but not between the 1-word and 2-word windows, *F*(19,31) = 1.14, *p* = .292, *η*_*p*_^*2*^ = .028.

**Table 2:** Mean reading time measures [in ms] for the three preview conditions.

Reading measure	1-word window		2-word window		Normal Reading	
	*M*	*SD*	*M*	*SD*	*M*	*SD*
TTRD	2483	(522)	2166	(587)	2250	(531)
GD	388	(76)	319	(79)	318	(73)
FFD	302	(40)	272	(46)	263	(41)

TTRD = Total trial reading duration; GD = Gaze duration; FFD = First fixation duration.

The amount of preview available had significant main effects on all three measures of reading time: FFD, *F*(2, 78) = 30.76, *p* < 0.01, *η*_*p*_^*2*^ = .441, as well as GD, *F*(1.86, 72.71) = 74.98, *p* < 0.01, *η*_*p*_^*2*^ = .658 and TTRD, *F*(1.73, 67.58) = 18.95, *p* < 0.01, *η*_*p*_^*2*^ = .327. Post-hoc analyses, corrected for multiple comparisons with *α*’ = .005, confirmed significant differences in all of these measures between the 1-word and 2-word window conditions, *F*(1,39) ≥ 28.02, *p* <. 001, *η*_*p*_^*2*^ ≥. 418, and between the 1-word window and the normal reading condition, *F*(1,39) ≥ 20.89, *p* <. 001, *η*_*p*_^*2*^ ≥. 349. However, there was no significant difference between the 2-word window and the normal reading conditions, *F*(1,39) ≤ 4.24, *p* ≥. 046, *η*_*p*_^*2*^ ≤. 098.

The preview benefit was measured as the difference between the 1- and 2-word window condition. Mean preview benefit was significant in all three measures; specifically, it was 29 ms in FFD, *t*(39) = 5.29, *p* <. 001, *d* = .837, 69 ms in GD, *t*(39) = 11.89, *p* <. 001, *d* = 1.881, and 319 ms in TTRD, *t*(39) = 7.32, *p* <. 001, *d* = 1.158.

Table 3 shows the split half reliabilities for the three reading time measures and the preview benefit. Although slightly less reliable than the eye movement measures for the underlying conditions alone, the preview benefit (as the difference between these conditions) has good reliability (>. 84), indeed much better than what one might expect for a difference score.

**Table 3:** Split-half reliabilities for the normal reading condition and the preview benefit.

Reading measure	Normal reading	Preview benefit (1-word minus 2-word window)
TTRD	.971	.849
GD	.953	.885
FFD	.993	.893

TTRD = Total trial reading duration, GD = Gaze duration, FFD = First fixation duration.

When averaged across all three reading conditions, the participant’s overall reading duration (TTRD) showed a significant negative correlation with the size of the preview benefit in the observed values *r* = -.33, *p* = .04, and also when based on the LMM, the correlation parameter between intercept (i.e., an estimate of overall reading speed) and the preview benefit (i.e., the effect of the contrast between 1-word and 2-word conditions) was estimated as *r* = -.35, <. 05); in other words, fast readers extracted more information from parafoveal vision. The correlation was also significant for the LMM of GDs (-.37) and in the same direction, but not significant for FFDs (-.27). Note that LMM correlation parameters are estimated simultaneously with all other model parameters. Thus, they are not computed on the basis of within-subject differences.

### Relationships between Extrafoveal Vision and Reading Measures

Table 4 summarizes the relationships between the three measures of vision (crowded letter identification, single-letter identification, and foveal acuity) with various measures of reading time and parafoveal preview benefit in the list reading task.

**Table 4:** Correlations between vision and reading measures.

Reading Measure	Condition	Crowding	Extrafoveal acuity	FrACT acuity
		*r* (*p*)	*r* (*p*)	*r* (*p*)
TTRD	Normal reading	-.17 (.14)	-.07 (.33)	. 01 (.94)
	1-word	**-.27 (.04)** *	-.23 (.07)	-.07 (.69)
	2-word	**-.30 (.03)** *	-.26 (.05)	-.06 (.70)
	Preview benefit	. 16 (.16)	. 15 (.18)	. 02 (.90)
GD	Normal reading	-.18 (.12)	-.10 (.28)	.01 (.93)
	1- word	-.21 (.09)	-.14 (.19)	-.17 (.30)
	2-word	-.25 (.06)	-.20 (.10)	-.15 (.37)
	Preview benefit	.13 (.21)	. 16 (.16)	.00 (1.00)
FFD	Normal reading	-.09 (.28)	-.11 (.24)	. 04 (.82)
	1- word	. 04 (.41)	-.08 (.31)	-.12 (.47)
	2-word	-.16 (.15)	-.22 (.08)	-.14 (.38)
	Preview benefit	. 13 (.43)	**. 34 (.01)** *	. 05 (.72)

*Note*. Preview benefit is the difference between the 2-word and 1-word moving window condition.

* Asterisks (*) indicate significant correlations at *p* <. 05 (one-tailed test).

#### Crowding

Across participants, correlations between the observed measure of crowding (i.e., the threshold eccentricities for flanked letters, averaged across left and right hemifield presentations and across all test repetitions) and TTRD were modest but significant, both for the 1-word window condition (*r* = -.27, *p* = .04) and the 2-word window condition (*r* = -.30, *p* = .03). For the normal reading condition, which also allowed for regressive saccades, the correlation with TTRD was not significant (*r* = -.17, *p* = .14). The two measures of fixation time, GD and FFD, did not correlate with crowding in any condition. There was, however a trend for GD in the 2-word condition into the same direction as for TTRD (*r* = -.25; *p* = .06).

There was no significant correlation between crowding and preview benefit (in TTRD, GD, or FFD), neither for the observed values, *r* ≤. 16, *p* ≥. 16, nor in the LMM-based estimates, *r* ≤. 17.

In a supplementary analysis, we tested whether higher correlations are obtained if crowding is operationalized not as the raw threshold eccentricity in the flanked letter condition, but as the difference between the measured thresholds with and without flanking letters (i.e., single letter threshold minus crowded threshold). Again, this difference measure was averaged over all conditions per participant (hemifields and test repetitions). In contrast to the raw crowded eccentricities, this difference measure showed no relationship with TTRD in any of the three reading conditions, neither in the observed values, *r* ≤. 08, *p* ≥. 30, nor in LMM-based correlation estimates, *r* ≤. 08. There was also no correlation with preview benefit.

#### Single letter identification

Despite a trend, correlation measures between single letter identification eccentricities and TTRD were not significant (-.26 < *r* <-.07; *p* >. 05). Single letter identification was uncorrelated with the preview benefit in TTRD, both in the observed measure (*r* = .15) and with the LMM-based estimate (*r* = .16).

In contrast, single letter identification correlated with the preview benefit (2-word minus 1-word condition) in FFD, both for the observed measure (*r* = .34; *p* = .01) and for the LMM-based estimate (*r* = .33). In contrast, there was no such correlation of single letter identification with mean FFD or with mean GD in any of the different preview conditions.

#### FrACT

Foveal acuity scores, measured with the Freiburg Acuity Test, did not correlate with any measure of reading speed.

## Discussion

The present study aimed at investigating the relationship between measures of reading speed and measures of extrafoveal vision on an individual differences level. We hypothesized that readers with better extrafoveal vision take up more information from parafoveal words during reading, leading to an overall higher reading speed. The main results of the present study are as follows: (1) Our eye-tracking experiment allowed us to reliably assess individual differences in reading behavior, including the size of the preview benefit. (2) With our adaptive test, we also reliably measured individual differences in extrafoveal acuity and crowding thresholds for peripheral letters. (3) As hypothesized, we found that faster readers show a larger preview benefit than slower readers, suggesting that they take up more information from the parafoveal word. (4) Importantly, we observed a modest but significant relationship between crowding and the overall speed of reading (TTRD) across individual readers in reading conditions with a one-word or two-word moving window. In contrast, crowding thresholds did not relate to the size of the preview benefit, although there was a mild correlation between preview benefit and uncrowded letter recognition (extrafoveal acuity). Next, we will discuss these findings in turn.

### Reading

Reading speed was assessed with a simplified procedure in which participants read lists of words from left to right at their own pace. This procedure allowed us to obtain not only an estimate of overall self-paced reading speed, but also of the magnitude of the preview benefit. For this purpose, we compared a condition without useful parafoveal information about the upcoming word (1-word window) with a condition that allowed such a preview (2-word window). The effects of this preview manipulation on eye movement measures, that is, on fixation durations, gaze durations, the overall reading duration for the trial, and the size of the preview benefit, were as expected. Importantly, individual differences in oculomotor behavior were again highly reliable. This held true also for the preview benefit despite the fact that it is computed as a difference score. Therefore, we conclude that our experimental setting yielded plausible and stable measures both of overall reading speed and its constituent processes, including preview benefit.

### Extrafoveal Vision

To assess extrafoveal vision, we used an adaptive test, which is based on established procedures of measuring the visual span [28]. Our procedure held the target-flanker spacing and letter size constant and instead manipulated eccentricity in order to determine individual differences in the size of the windows for uncrowded or crowded letter identification. Furthermore, we only varied stimulus eccentricity on the horizontal meridian, which is relevant for normal reading, without testing other regions of the visual field. Results of this procedure suggest that crowded letters could only be identified parafoveally, that is, at eccentricities of less than 5°. As expected from the literature, threshold eccentricities were larger in the right than in the left visual field. Recognition thresholds for single letters, measured in the same way as for crowded letters (merely omitting the flanking letters) was possible far into in the peripheral visual field with mean threshold eccentricities of 10–11°.

Importantly, across three testing sessions, reliabilities for uncrowded and crowded letter identification were good to excellent. Therefore our adaptive procedure seems to provide reliable and valid threshold measures of letter identification in extrafoveal vision.

### Relationship between Reading and Extrafoveal Vision

We held two primary hypotheses for our study. First, we expected that crowding would impose a limit on reading speed and hence that the individual threshold for the successful identification of crowded letters should be related to individual reading speed measures. Second, it was assumed that extrafoveal visual acuity (in the absence of crowding letters) might also contribute to reading speed. Both, crowded and uncrowded letter recognition might also be related to the size of the processing benefit obtained from having a preview of the upcoming word.

#### Crowding

In line with these expectations, thresholds for crowded letter identification correlated with TTRD, used here as a global measure of reading speed. This correlation was modest and only significant in the two experimental conditions with a gaze-contingent moving window (1-word and 2-word window). Importantly, these conditions force readers to recognize each word during first-pass reading, because all words to the left of the current fixation are again covered by a mask. In contrast, the relationship between crowding and reading speed did not reach significance in the normal reading condition without a moving window. In this condition, all words in the list remained visible troughout the trial, meaning that it was possible for the reader to return to earlier words in the list. In fact, in this condition, 32% of all trials (SD = 19.4%) contained at least one regressive saccade to an earlier word in the list. The percentage was much lower in the 1-word and 2-word condition (M = 6.7%, SD = 9.4% and M = 12.1%, SD = 11.9%, respectively). The absence of a significant relationship between crowding and TTRD in the normal reading condition might therefore be explained by the additional variance in TTRD generated by re-reading behavior.

From previous findings, for example by Pelli, Tillman, Freeman, Su, Berger and Majaj [29] one might have expected a much stronger relationship between crowding and self-paced reading speed. However, the modest size of the relationship between reading speed and extrafoveal vision observed here is fully in line with a recent report by Risse [51] who correlated the visual span profile for crowded letter recognition with reading speed, measured as reading time per word in a natural sentence reading task. Despite marked differences in quantifying both reading speed and crowding in the study by Risse, the relationship was very similar to that found in the present study. Taken together, the two studies suggest that the relationship between crowding and the speed of saccadic reading is significant but modest in size. We find converging evidence, even though paradigms for both variables differed between the studies (reading speed: syntax-free word lists versus normal sentences and crowding: threshold versus visual span profile). A possible concern regarding the present results is that we used a different font size and letter spacing in the EVA and in the reading experiment. Due to this fact, the thresholds (or window sizes) for letter identification determined in the EVA cannot be direcly mapped onto the number of letters previewed during saccadic list reading. However, such a 1:1 mapping was not possible anyway due to the criterion of 65% correct responses in the adaptive EVA (e.g., a less conservative criterion would have yielded larger window sizes for letter identification). For the correlative approach of the present study it is not important to measure window size in absolute terms, but to obtain reliable estimates of the relative sizes of the windows of different participants. As suggested by the sizeable reliablities, this was successful.

We offer three possible explanations why the relationship between crowding and saccadic reading speed is not as strong as it might be expected from other reports [27, 29]. First, in several previous studies, reading speed was operationalized as identification threshold for words presented at a given eccentricity while crowding was varied within-participant by changing the target-flanker spacing [27, 28, 52]. While such a procedure tests the limits of the system (its maximum performance), it may be less informative about self-paced reading with eye movements under everyday conditions. Furthermore, crowding is of course highly unlikely to be the only source of individual differences in the speed of normal reading. For example, the likelihood of regressive saccades is both a stable individual characteristic and a major contributor to reading speed [5]. During normal reading, regressions are triggered both by oculomotor errors (correction of saccadic overshoot) and problems related to sentence comprehension. For example, when a disambiguating word in a sentence enforces a reinterpretation of the preceding sentence structure, readers typically trigger a regression to re-read the sentence and correct the incorrect expectation. Although the context-free word lists of the present study did not contain syntactic or semantic ambiguities, we found that subjects nevertheless executed regressive saccades, presumably to resolve uncertainty about the presence of a target word at earlier list positions (“was the last word really no animal?”). As discussed above, this may explain why crowding contributed significantly to reading speed only in the 1- and 2-word window condition, that is, under conditions where the usefulness of regressions to previous words was restricted or absent. When regressions were possible, the relationship between reading speed and crowding dropped below significance.

Second, in contrast to most previous studies about effects of crowding on reading speed, we were interested in correlations across individuals rather than experimental conditions. As mentioned above, experimental effects cannot simply be transferred to individual differences. Crowding might be considered a relatively pure facet of extrafoveal vision, whereas natural reading—even of syntax-free word lists—is a complex skill. Thus, individual differences in this skill are the result of multiple sources, such as amount of print-exposure, vocabulary knowledge, basic word recognition performance [53, 54], and working memory capacity [55–60]. These additional sources of variance may limit the relative contribution of crowding to reading speed.

Third, and in contrast to most previous investigations on crowding and reading speed, our procedures allowed for (and required) eye movements. Recent studies have reported that the execution of a saccade towards a crowded stimulus [34] or the accompanying shift of visuospatial attention [36] tend to release the stimulus from crowding in a brief time window prior to saccade onset. Hence, it is possible that in normal reading situations, which involve the planning of saccades to upcoming words, crowding plays a smaller role than in experiments where reading rate estimates are based on the passive presentation of words during fixation (RSVP). Nevertheless, experiments with flash card reading (presentation of a whole sentence in four short lines that is read with eye movements) by Yu, Cheung, Legge and Chung [27] and the present data show that a relationship is still observed.

#### Extrafoveal Acuity

Contrary to our expectations, we found no correlation between crowding and the size of the preview benefit. However, a modest but significant relationship was found between extrafoveal acuity (uncrowded letter recognition) and the preview benefit in first fixation durations: Participants with a wider field of high-acuity vision showed a larger preview benefit. Although such a result seems to be plausiple, to our knowledge, it has not been previously reported. Notably, a significant relationship was found only in first fixation duration, but neither gaze duration, nor total trial reading duration and the correlations seem to decrease for”late”fixation time measures. Thus, whereas there seems to be a small initial advantage for readers with higher acuity, this advantage appears to be diluted by high-order, cognitive processes in other, more complex reading time measures, including the overall speed of reading (TTRD). Still, the positive relationship between preview benefit and extrafoveal acuity offers some interesting perspectives for reading research. In particular, extrafoveal acuity might be an interesting covariate for future research on the preview benefit.

### Relationship between Preview Benefit and Reading Speed

In line with our hypothesis that parafoveal information contributes to reading speed, reading durations (TTRD) were shorter for participants with larger preview benefit. This correlation between reading speed and preview benefit confirms previous findings about differences in preview benefit between groups of slow and fast readers [10, 11], and extends them by demonstrating a linear relationship across participants with an unselected range of variability in reading speed.

Such a linear relationship was also reported by Risse [51] but it was of opposite direction. Unexpectedly, in her study, slow readers showed a larger preview benefit than fast readers for high-frequency words. The most likely source of this difference are the differences in experimental paradigms and resolving this difference will require additional work. Both studies failed to detect an expected significant relation between crowding and preview benefit. At an intuitive level, this null result is quite surprising and also suggests that the relationship between reading speed, crowding, and preview benefit is more complex than assumed.

### Reading speed, preview benefit and crowding

A speculative proposal to reconcile these results contains the assumption that preview benefit and crowding relate to different processes, which contribute independently to reading speed. Whereas there was no significant relationship between crowding and early fixation time measures (FFD, GD), crowding correlated with overall reading speed (TTRD). Surprisingly, this relationship was even found in the reading condition with a 1-word window, in which the reader obtains no useful preview on the upcoming word, because this word is masked by x-letters. Consequently, this relationship cannot be mediated via the uptake of information about the letters or shape of the upcoming word. Instead, this result strongly suggests that crowding is not only detrimental for parafoveal word identification, but possibly also affects other reading-related processes that contribute to overall reading speed. For example, coarse resolution of attention [25] or unfocussed spatial attention [26] associated with crowding might interfere with the shift of attention towards the next word or with the programming of a precise saccade that lands at the optimal viewing position of the crowded string [61].

### Conclusions

Previous research documented intraindividual relations between crowding and reading speed. In the present study we investigated this relationship with regard to interindividual differences. We found a significant but modestly positive correlation between the speed of self-paced list reading and the size of the uncrowded window and also a positive correlation between reading speed and preview benefit. We failed to find evidence for the expected correlation between the uncrowded window and preview benefit. Such an absence of expected evidence is of course no evidence of its absence [62] but should encourage further research to reconcile theoretical expecations and empirical results about interindividual differences in experimental effects.
